# Inflammatory Rheumatic Disorders and Bone

**DOI:** 10.1007/s11926-012-0252-8

**Published:** 2012-04-03

**Authors:** Irene E. M. Bultink, Marijn Vis, Irene E. van der Horst-Bruinsma, Willem F. Lems

**Affiliations:** 1 Department of Rheumatology, VU University Medical Center, De Boelelaan 1117, 1081HV Amsterdam, The Netherlands; 2 Department of Rheumatology, Erasmus Medical Center, Dr. Molewaterplein 50-60, 3015 GE Rotterdam, The Netherlands; 3 Department of Rheumatology, VU University Medical Center and Reade, De Boelelaan 1117, 1081HV Amsterdam, The Netherlands

**Keywords:** Osteoporosis, Fractures, Rheumatoid arthritis, Systemic lupus erythematosus, SLE, Ankylosing spondylitis, Metabolic bone disease, Inflammation, Bone

## Abstract

Inflammatory joint diseases such as rheumatoid arthritis, as well as other rheumatic conditions, such as systemic lupus erythematosus (SLE) and ankylosing spondylitis, comprise a heterogeneous group of joint disorders that are all associated with extra-articular side effects, including bone loss and fractures. The concept of osteoimmunology is based on growing insights into the links between the immune system and bone. The pathogenesis of osteoporosis in these patients is multifactorial. We have, more or less as an example, described this extensively for patients with SLE. High disease activity (inflammation) and immobility are common factors that substantially increase fracture risk in these patients, on top of the background fracture risk based on, among other factors, age, body mass index, and gender. Although no fracture reduction has been shown in intervention studies in patients with inflammatory rheumatic diseases, we present treatment options that might be useful for clinicians who are treating these patients.

## Introduction

**Fig. 1:**
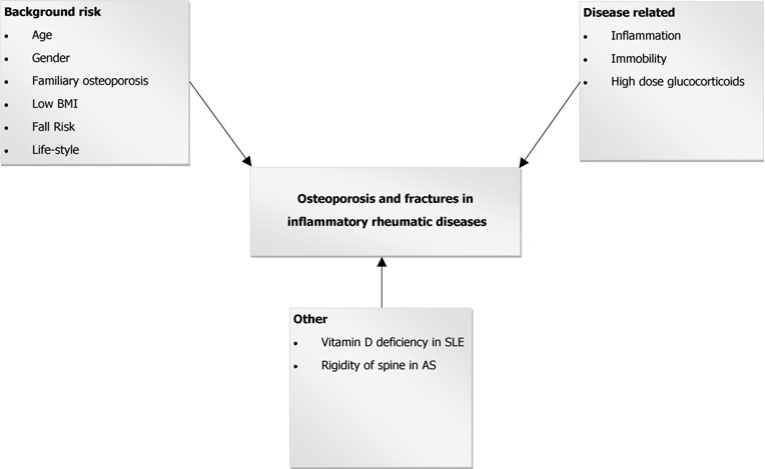
Risk factors for osteoporosis and fractures in inflammatory rheumatic diseases. *AS* ankylosing spondylitis; *BMI* body mass index; *SLE* systemic lupus erythematosus

Osteoporosis-related fragility fractures represent one of the most important complications that may occur in patients with rheumatic diseases; obviously, these fractures may contribute to an important decrease in quality of life. Disease activity (inflammation), immobility, and treatment with glucocorticoids are the main factors that increase the risk of osteoporotic fractures, on top of the background fracture risk based on, among other factors, age, body mass index, and gender (Fig. 1). Recent data in the field of osteoimmunology, the cross-talk between cytokines and bone, have elucidated that activated inflammatory cells at sites of inflammation produce a wide spectrum of cytokines that stimulate local and generalized bone resorption, and that inhibit (in rheumatoid arthritis [RA]) or stimulate (in ankylosing spondylitis [AS]) bone formation. We discuss extensively the complex pathogenesis of generalized bone loss in systemic lupus erythematosus (SLE), followed by a description of bone involvement in RA and, somewhat briefly, in AS. Finally, treatment options are discussed.

## Osteoporosis and Fractures in Systemic Lupus Erythematosus

SLE is a chronic autoimmune disease that usually affects women in their reproductive years. Over the past decades, the survival of SLE patients has improved dramatically, and the morbidity pattern has shown a shift toward long-term disease complications, including generalized osteoporosis (fractures).

Osteopenia, defined as a T-score in the lumbar spine and/or hips between −1 and −2.5, is demonstrated in 25 % to 74 % of SLE patients [1, 2]. Osteoporosis, a T-score less than −2.5, is reported in 1.4 % to 68 % of patients [3, 4]. These wide variations are probably related to differences in study design, size, age, ethnicity, sex, disease severity, and medication use between the investigated patients. The etiology of bone loss in SLE is supposed to be multifactorial, including traditional risk factors for osteoporosis such as old age and low body weight, but also incorporates disease-related factors: inflammation, metabolic factors, hormonal factors, serologic factors, and medication-induced adverse effects [5•].

The traditional risk factors, age [6, 7], postmenopausal status [5•, 6], low body weight [7], or low body mass index [6–8], have all been recognized as independent risk factors for osteoporosis in SLE.

The influence of gender on bone loss in SLE is unclear, as the majority of studies investigated (almost) exclusively female patients. In line with findings in the general population, two studies in lupus patients showed that white [9] or non-African Caribbean ethnicity [10] was a risk factor for osteoporosis, while another study demonstrated reduced bone mineral density (BMD) in African American women with SLE compared with white patients after controlling for clinical variables and after adjusting for glucocorticoid (GC) use [11]. Smoking is not reported as a risk factor for osteoporosis in SLE [6, 8, 10] but has recently been identified as a risk factor for osteoporotic fractures in a study from the Hopkins Lupus Cohort [12].

Chronic systemic inflammation may contribute to bone loss in SLE. In patients with active lupus, increased serum levels of tumor necrosis factor (TNF) [13] and oxidized low-density lipoprotein (LDL) [14] were demonstrated. Oxidized lipids induce activation of T cells, which in turn induce increased production of receptor activator of nuclear factor-κB ligand (RANKL) and TNF. Both TNF and RANKL enhance the maturation and activity of osteoclasts [13]. In addition, oxidized LDL may negatively influence bone formation by reducing osteoblast maturation [15]. In premenopausal women with recently diagnosed and untreated SLE, decreased serum levels of osteocalcin (marker for bone formation) and increased cross-links excretion in the urine (marker for bone resorption) were demonstrated [16].

Until now, clinical studies have failed to demonstrate a relationship between disease activity score and bone loss in SLE [6, 8, 9, 17], which might be explained by the cross-sectional design of these studies. However, low complement C4 levels (a measure of active disease) were a predictor of low spine BMD in the Hopkins Lupus Cohort [7]. Moreover, several studies reported an association between organ damage and reduced BMD [6, 9, 17, 18]. Because organ damage accumulates in patients with prolonged active disease, this finding suggests that disease activity negatively influences BMD in SLE.

Although inflammation-induced lupus nephritis may occur in 50 % to 60 % of the SLE patients ever during the disease course, only one study in older female SLE patients reported an association between impaired renal function and low BMD [6], probably related to the exclusion of patients with renal failure in most of the studies.

Studies in SLE patients in different geographical regions demonstrate an increased frequency of vitamin D deficiency [19–21], a metabolic condition that induces bone loss. Moreover, low 25-hydroxyvitamin D (25[OH]D) serum levels were associated with low spine BMD in Dutch lupus patients [8].

Vitamin D status might be negatively influenced by several factors in SLE: photosensitivity (leading to avoidance of sun exposure) and use of sunscreens, dark skin pigment, renal failure, GC use, and probably hydroxychloroquine (HCQ) use. In SLE patients, an association between cumulative GC use and low levels of both 25(OH)D and 1,25(OH)_2_D has been reported [21].

The antimalarial HCQ, which is frequently used to treat SLE, might impair vitamin D status, as this drug is supposed to inhibit the conversion of 25(OH)D to 1,25(OH)_2_D by inhibiting hydroxylase α1. A cross-sectional study demonstrated reduced 1,25(OH)_2_D levels in HCQ-treated SLE patients compared with nonusers [20]. However, another study reported higher 25(OH)D levels in patients treated with HCQ [22]. In two cross-sectional studies in female patients, HCQ use was associated with higher BMD in the spine [2, 9] and hip [9]. Further longitudinal studies in large groups of SLE patients as well as in patients with other diseases treated with HCQ are needed to unravel the relationship between HCQ use and bone (metabolism).

Hormonal changes in patients with SLE may adversely affect bone mass. SLE is characterized by a relatively high estrogenic and low androgenic state, and a decrease in dehydroepiandrosterone (DHEA) and an association between low DHEA sulfate levels and low BMD in SLE patients was reported [23].

A study on the relationship between serologic factors and BMD demonstrated that the presence of anti-Sm and the absence of anti-Ro were associated with a higher spine BMD [2].

GCs are frequently used in SLE for the treatment of disease exacerbations or complications and play a dual role with respect to bone mass. On one hand, GCs induce bone loss, but on the other hand, they have a beneficial effect on bone mass by suppressing inflammation. The several cross-sectional studies on the relationship between GC use and BMD in SLE have yielded conflicting results. However, three of four small longitudinal studies performed showed increased bone loss in all patients receiving GC treatment [3], or exclusively in patients treated with more than 7.5 mg prednisone daily [24, 25].

Because changes in BMD are usually not associated with clinical signs and symptoms, fracture data are clinically more relevant. In a population-based study in women with SLE, the incidence of clinical fractures was nearly fivefold increased compared with healthy controls [26]. Symptomatic fractures are reported in 6 % to 12.5 % of the patients because lupus diagnosis [7, 10, 26, 27] and age [7, 10, 26], postmenopausal status [7], smoking [12], disease duration [27], renal failure [12], Raynaud’s phenomenon [12], reduced BMD [10], and presence of lupus anticoagulant [12] are identified as risk factors.

Moreover, GC treatment is associated with future osteoporotic fractures [7], and older age at lupus diagnosis and duration of GC treatment are associated with time from lupus diagnosis to fracture [26]. Prevalent vertebral fractures are demonstrated in 20 % to 26.1 % of the SLE patients [8, 17, 28]. Age [17, 28], low BMD [17, 28], previous use of intravenous methylprednisolone [8], male sex [8], and, surprisingly, high body mass index [28] were associated with vertebral fractures. Importantly, two studies demonstrated normal BMD in 29 % to 35.8 % of the patients with one or more vertebral fractures [17, 28], which illustrates the limited value of BMD measurement in the assessment of future fracture risk.

## Osteoporosis and Fractures in Rheumatoid Arthritis

RA is characterized by the presence of inflammatory synovitis and the destruction of cartilage and bone. These bone complications are very characteristic of RA and can be divided into three different forms: periarticular bone loss adjacent to the inflamed joints, bone erosions, and systemic osteoporosis. Remarkably, the local and generalized bone loss share common pathways: the RANKL/osteoprotegerin (OPG) pathway [29]. The RANKL/OPG pathway is involved in the regulation of bone resorption in RA by stimulating the activation, differentiation, and proliferation of osteoclasts by RANKL, with OPG acting as a decoy receptor [30]. Recently, it has been elucidated that several inflammatory cytokines, such as TNF-α, interleukin (IL)-1, IL-6 and IL-17, upregulate RANKL, with subsequent activated osteoclastogenesis [31•, 32••]. RANKL is expressed by osteoblasts but also by activated T cells and B cells and seems to be a critical factor for joint destruction in RA. Interestingly, in a study in RA patients in which all patients were treated with methotrexate (MTX), treatment with denosumab, a monoclonal antibody against RANKL, versus placebo, reduced joint damage [33]. When looking more in detail, the erosion score was lower in both the 60-mg and 180-mg denosumab-treated patients, while the joint space narrowing was unattached. Although there are some suggestions that joint space narrowing is more closely related to physical dysfunction, it is important to realize that nowadays, many RA patients are treated with the effective but costly combination of MTX and a biological (eg, a TNF blocker), and that the combination of high-dose MTX plus denosumab could be an attractive alternative. However, this should be investigated further.

The Wnt pathway is a regulatory pathway of osteoblast activity. At the molecular level, the activation of the Wnt/B catenin pathway is crucial for osteoblastic differentiation [34, 35]. Two blockers of the Wnt-signaling pathway that play an important role in RA are dickkopf-1 (Dkk-1) and sclerostin. TNF-α, for instance, can induce both sclerostin and Dkk-1 [32••]. Garnero et al. [36] found in a study in early RA that patients with Dkk-1 levels in the highest quartile had a more than five times higher relative risk for radiological progression than patients in the lowest quartile. In another study in RA patients, RANKL/OPG was lower than in healthy controls, while Dkk-1 and sclerostin were higher. After treatment with anti–IL-6, OPG/RANKL increased, Dkk-1 decreased, and sclerostin increased [37]. Xu and colleagues [38] investigated osteoimmunology in the pathogenesis of osteoporosis in 64 hospitalized disease-modifying antirheumatic drug (DMARD)-naive RA patients and age- and sex-matched healthy controls. They found a higher prevalence of osteoporosis (T-score < −2.5), increased levels of RANKL, and decreased OPG levels in RA patients compared with healthy controls. In fact, these data illustrate the elevated bone resorption but also the reduced bone formation in patients with inflammatory rheumatic diseases, as has been reviewed by Schett et al. [39].

Even a small rise in the level of systemic inflammation can precipitate bone loss and may lead to fractures [32••]. The prevalence of osteoporosis in RA is increased about twofold compared with the general population: this was among other findings shown in a study by Haugeberg et al. [40], in which the prevalence of osteoporosis, defined as a T-score less than −2.5 in females, was increased 2 times in 394 female RA patients compared with a reference population [40]. Before the introduction of biologicals, a high bone loss was observed in a longitudinal study in early RA: −2.4 % at the spine and −4.3 % at the hip [41]. In a subgroup analysis, bone loss in both the spine and the hips was much larger in those patients with high C-reactive protein (CRP) levels (>20 mg/dL) than in those patients with low CRP levels (<20 mg/dL) (eg, in the spine, −2.1 % vs 0.2 %, respectively). The same was found in the lumbar spine for patients with low functional capacity (Health Assessment Questionnaire [HAQ] score >1) compared with patients with a better HAQ score (<1) (−1.9 % vs −0.2 %, respectively). Although bone loss was substantial in earlier studies, we have recently shown that treatment with anti-TNF arrests BMD loss at the hip and the spine. In an open cohort study of 102 RA patients from Norway and The Netherlands, all treated with infliximab, there was no bone loss at the spine and hip after 1 year, while BMD in patients with a European League Against Rheumatism (EULAR) good response showed a favorable change in BMD compared with patients not achieving such a response, indicating that adequately suppressing the inflammation in RA is beneficial for the prevention of generalized bone loss [42]. In the BEST study, a similar result was shown. The BEST study is a randomized controlled trial comparing four different treatment strategies, including, among others, COBRA and MTX/infliximab, in early RA patients striving for remission. In all four groups, there was only moderate generalized bone loss at 2 years at the hip and spine (−0.5 % to 1.0 %) [41]. In addition, patients in remission had less bone loss than patients with low or moderate disease activity (0 %, −2 %, and −3 %, respectively) [42]. However, in both studies [43, 44], BMD of the hand measured by digital x-ray radiogrammetry was not arrested but showed a significant decrease, indicating that anti-inflammatory treatment still needs some improvement.

It is important to realize that not low bone mass, but fractures are the clinically most relevant outcome. Earlier studies have shown that patients with RA are also at an increased risk of both vertebral and nonvertebral fractures [45, 46]. In a large cohort study from the General Practice Research Database, it was shown that the risk of osteoporotic fractures in RA patients is increased 1.5-fold (1.4–1.6) compared with healthy controls [47]. The most frequently reported risk factors associated with osteoporosis in RA are inflammation, immobility, corticosteroid use, and disease duration, but traditional risk factors for osteoporosis (ie, low BMD and previous fractures) also contribute to fracture risk in RA [48••].

In a recent study, we showed that in a 5-year follow-up study in female RA patients older than 50 years of age with established disease, new nonvertebral fractures occurred in 16 % of patients, and a new radiological vertebral fracture occurred in 19 % of the patients [49]. Compared with historical controls, these frequencies are about 1.5 to 2 times higher than expected. Recently, Amin and colleagues [50] performed a retrospective cohort study in RA patients and matched healthy controls. They found that the risk of osteoporotic fractures was particularly increased in younger RA patients. In the whole group of female RA patients, the OR for an osteoporotic fracture was 1.7 (95 % CI, 1.4–2.2), but in the subgroup of female patients younger than 50 years of age, an OR of 4.3 (95 % CI, 2.4–7.8) was found.

## Osteoporosis and Fractures in Ankylosing Spondylitis

AS is a chronic inflammatory disease with an elevated risk of vertebral fractures. More than 20 years ago, it was documented that vertebral fracture risk is six to seven times higher than in healthy controls [51, 52]. In contrast, no data are available that have documented an elevated nonvertebral fracture rate in AS, which could be a true phenomenon, but an elevated fracture risk could also be masked by an inadequate design or a lack of power of the studies. It is important to realize that vertebral fractures in patients with AS are often associated with neurological signs and symptoms [53].

What is the underlying mechanism for the elevated fracture risk in AS? It is thought that it is probably a result of bone loss and/or elevated rigidity of the spine. Indeed, in several cross-sectional studies, the markers of bone resorption, such as the pyridinolines but also RANKL, were upregulated in cohorts of AS patients compared with healthy controls [54, 55]. Studies on BMD show a different pattern: in early AS, the BMD both at the spine and at the hips is lower than in healthy controls, while in contrast, in later stages, a further decrease in hip BMD can be found, whereas in the lumbar spine, the BMD might be increased due to the formation of syndesmophytes [56, 57]. Recent data suggest a low BMD and a high prevalence of vertebral fractures, even in patients with early spondyloarthropathies [58, 59]. As the bone loss in AS is related to inflammation, the proof of the pudding is in the eating: when patients are adequately treated with TNF-blocking agents, the usually occurring bone loss can be arrested [60]. Another point is the debate surrounding inflammation and syndesmophyte formation. Maksymowych et al. [61] observed that lesions on MRI predict syndesmophytes, while Schett [62] argued for an independent development.

Prevention of fractures in patients with AS is relatively simple in those with clinical risk factors for osteoporosis and a low T-score (<−2.5)—in other words, when there is an indication for anti-osteoporotic treatment irrespective of the presence of AS. In AS patients with a BMD in the osteopenic range, treatment decisions are more complicated.

## Prevention and Treatment of Osteoporosis and Fractures in Patients with Rheumatoid Arthritis, Systemic Lupus Erythematosus, and Ankylosing Spondylitis

General lifestyle measures are important for all patients with rheumatic diseases: an adequate calcium intake, prevention of falls, adequate vitamin D levels, and prevention of immobilization, if possible. Special attention must be paid to sufficient serum 25(OH)D serum levels in SLE patients because of photosensitivity. In addition, the prescription of adequate immunosuppressive medication to reduce inflammation-induced bone loss is important, which has been documented in RA.

It is important to realize that the relative risk of fractures is increased in all three patient groups, and thus, the absolute fracture risk is particularly high in those patients with a high background fracture risk (eg, postmenopausal women with an inflammatory rheumatic disorder). Unfortunately, intervention studies demonstrating the effectiveness of one of the available anti-osteoporotic drugs (eg, bisphosphonates) for fracture reduction in patients with RA, SLE, or AS have not been performed yet. However, the effectiveness of several anti-osteoporotic drugs (bisphosphonates, strontium ranelate, selective estrogen receptor modulators, denosumab, and teriparatide/PTH) has been clearly demonstrated in postmenopausal women with primary osteoporosis, which is a strong argument to prescribe these drugs in patients with inflammatory rheumatic disorders, particularly in those with high background fracture risk and moderate or high disease activity. Bisphosphonates (alendronate, risedronate, and zoledronate) are usually the first choice because they are widely prescribed, generally safe, and effective, even in the prevention of hip fractures, although there are some concerns about long-term safety [63].

Bisphosphonates are recommended for the prevention and treatment of GC-treated individuals without renal failure, but these drugs should not be prescribed to premenopausal patients planning a pregnancy, as bisphosphonates are associated with fetal abnormalities in animal studies [64]. Bisphosphonates should only be prescribed to premenopausal patients with (high risk of) severe osteoporosis, who have completed their families, or who have decided not to become pregnant for several years. Another point is the use of bisphosphonates during long-term use of GCs. Although bisphosphonates are effective in the initial phase of treatment, their use in long-term treatment can be criticized [65].

Denosumab, a monoclonal antibody against RANKL, is an attractive new therapeutic agent for osteoporotic patients with renal failure and for RA patients. Not only has an increase in BMD of the spine and the hips been demonstrated in RA patients, but also a strong reduction in joint erosions.

The situation is somewhat more complicated in patients with AS, as local bone loss and local new bone formation can be found in the same patient. Obviously, this makes it more difficult to decide which drugs to prescribe; in other words, collaborative studies elucidating the pathogenesis and new intervention studies are urgently needed in these patients.

## Conclusions

Several, if not all, inflammatory rheumatic diseases might be complicated by elevated bone loss and increased fracture rate. We focus on RA, SLE, and AS because bone loss and fracture rate are relatively adequately documented in these diseases. Several factors play a role in the fracture rate in these patients, varying from demographic factors such as age and body mass index to immobility and high disease activity. Recent data in the field of osteoimmunology, the cross-talk between inflammatory cells and bone cells, have provided some insight into the complex pathogenesis of bone loss in systematic inflammatory diseases. Fundamental studies have elucidated that the upregulated RANKL, with subsequent activated osteoclastogenesis, is an important determinant of bone loss in RA.

Clinical studies have demonstrated that adequate immunosuppressive therapy (eg, according to the treat-to-target principle) prevents both local and generalized bone loss. Thus, optimal treatment of the underlying condition is the first step toward prevention of fractures in these patients (eg, for RA, this has been documented for the use of TNF blockers or combination therapy with conventional drugs). Apart from that, a healthy lifestyle (calcium, vitamin D, prevention of falls, and immobilization) is important; for those patients with a low T-score, treatment with bisphosphonates or denosumab might be attractive.
